# Supplementary material for: Artificial relativistic molecules
Source: Nat Commun. 2020 Feb 10;11:815. doi: 10.1038/s41467-020-14635-z (PMC7010783; doi:10.1038/s41467-020-14635-z)
Supplement: Supplementary file 1 — Supplementary Information [file 41467_2020_14635_MOESM1_ESM.pdf]

Supplementary Information for “Artificial Relativistic Molecules”

Park et al.

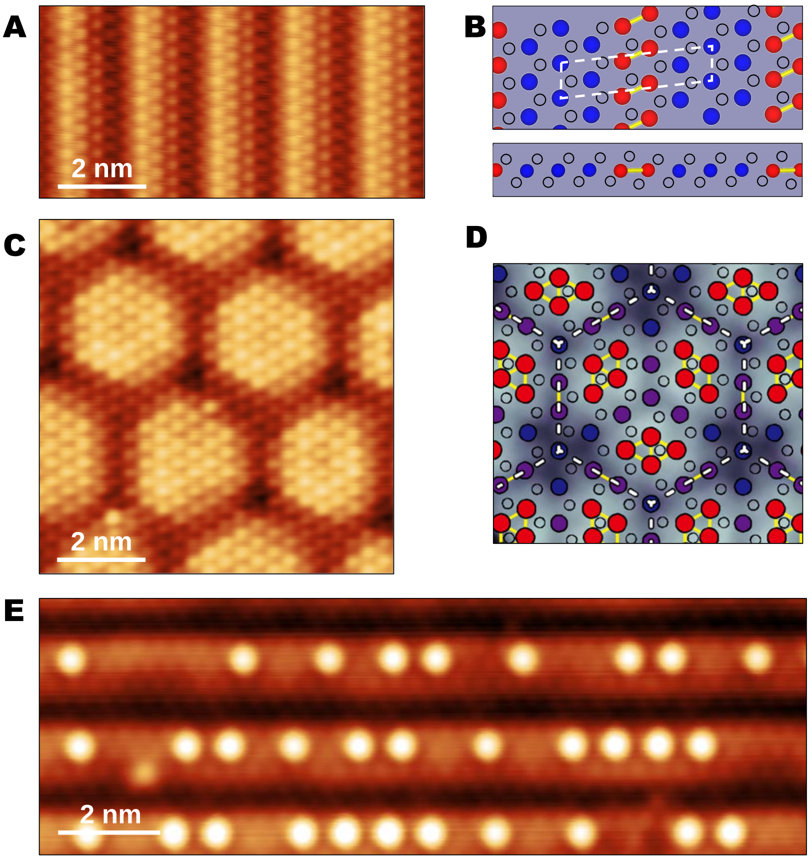

Supplementary Fig. 1: **Site selective Pb adsorption on the dimerized $IrTe_2$ substrate.** **a** STM image (V_s = 5 mV, I_t = 1 nA) and **b** dimerized structural model of the stripe phase (top and side views). **c** STM image (V_s = 10 mV, I_t = 1 nA) and **d** dimerized structural model of the honeycomb charge order phase. The STM topographies show the charge ordering induced by the dimerization of the Ir atoms. The dimerized Ir atoms (red circles) has $5d^{4+}$ valence electrons and others has $5d^{3+}$ valence electrons [1, 2]. **e** STM image (V_s = 5 mV, I_t = 3 nA) of the Pb atoms on the stripe phase. The Pb atoms selectively adsorb on the dimerized Ir site of the stripe phase. The atomic position of stripe phase in **b** and structural image in**d** were taken from Ref.[1] and [2], respectively.

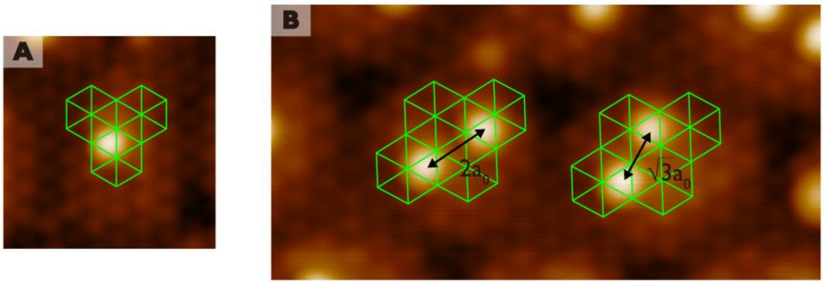

Supplementary Fig. 2: **Pb absorption site and Pb-Pb distance.** **a** Pb monomer and **b** Pb dimers. Green solid lines denote the Te-(1×1) lattice.

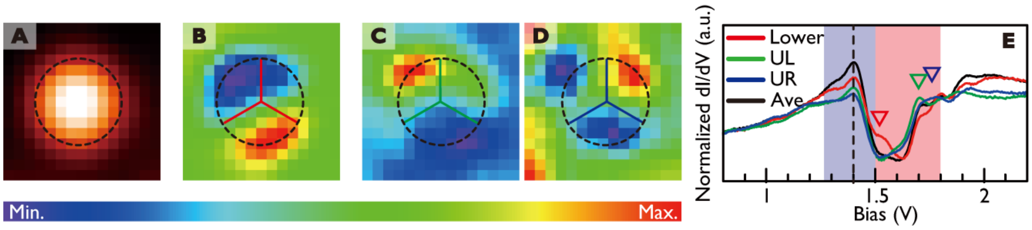

Supplementary Fig. 3: **Spatial dependence of the STS spectra.** **a** topography. **b-d** The dI/dV maps obtained at 1.52, 1.70 and 1.77 eV, respectively. **e** STS spectra of lower, upper left and upper right side of Pb single atom.

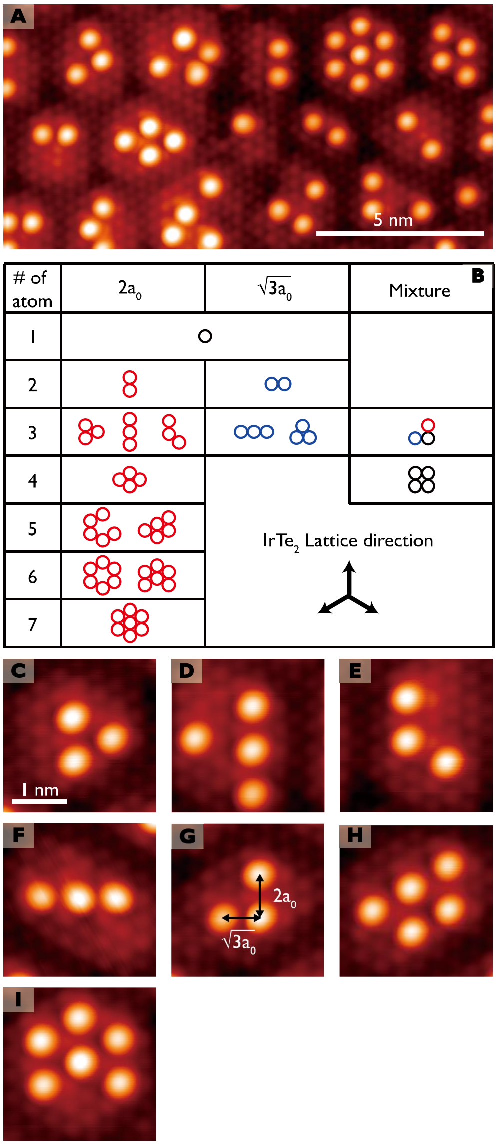

Supplementary Fig. 4: **STM topography and summary of all kinds of Pb clusters on the $IrTe_2$ hexagonal phase at 4.3 K.** **a** STM image of Pb clusters on atom-resolved hexagonal phase. **b** A summary of Pb clusters depending on the bonding length and the number of composed Pb atom. The $IrTe_2$ lattice symmetry is shown on lower right side. **c-i** Pb clusters of other configuration which is not included in Fig. 1 in text.

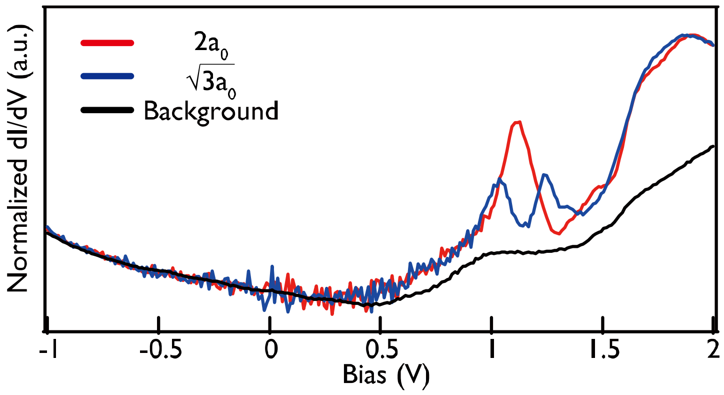

Supplementary Fig. 5: **Wide STS spectra of 2a$_0$ and $\sqrt{3}$a$_0$ spacing Pb dimers.** Peak energy is slightly shifted with Fig. 2 in text. The energy position depends on the charge doping concentration caused by Pb coverage. Red, blue and black curves are corresponding to the 2a$_0$ and $\sqrt{3}$a$_0$ spacing Pb dimers and background hexagons.

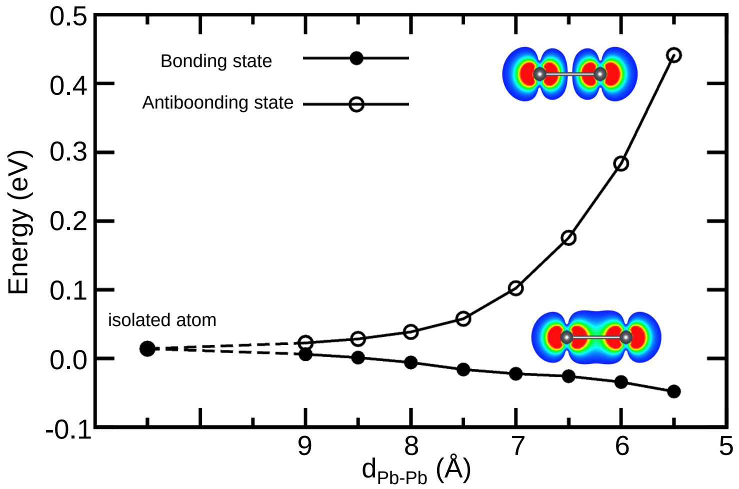

Supplementary Fig. 6: **Bonding/antibonding energy splitting of σ bond of the freestanding Pb dimer as a function of Pb-Pb distance.**

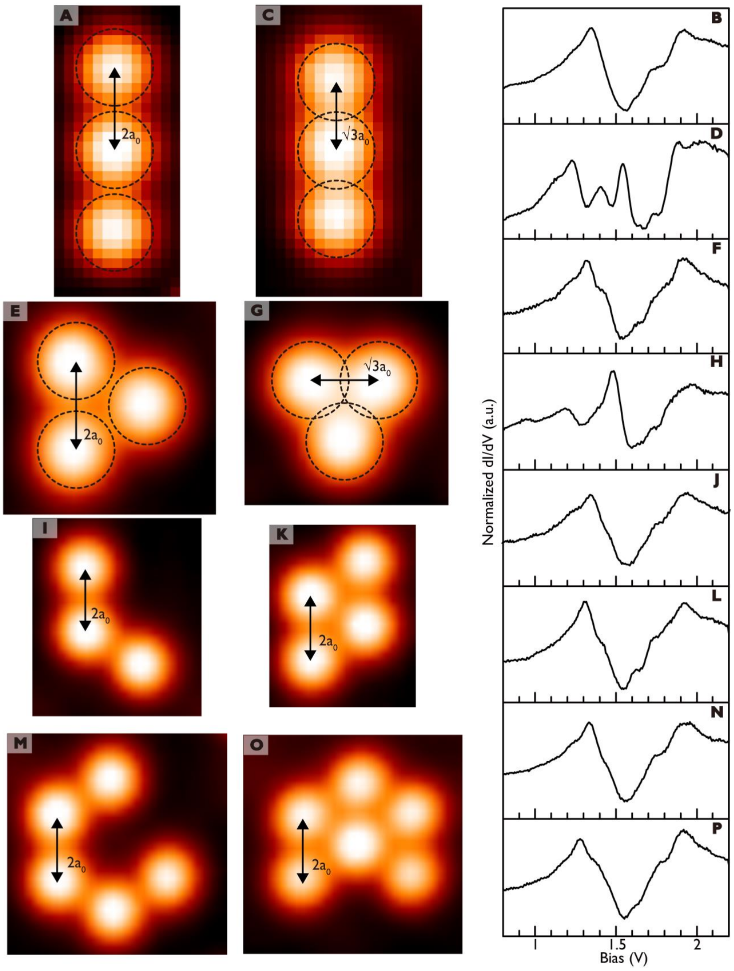

Supplementary Fig. 7: **Topography and STS spectra of the Pb molecules on $IrTe_2$.**

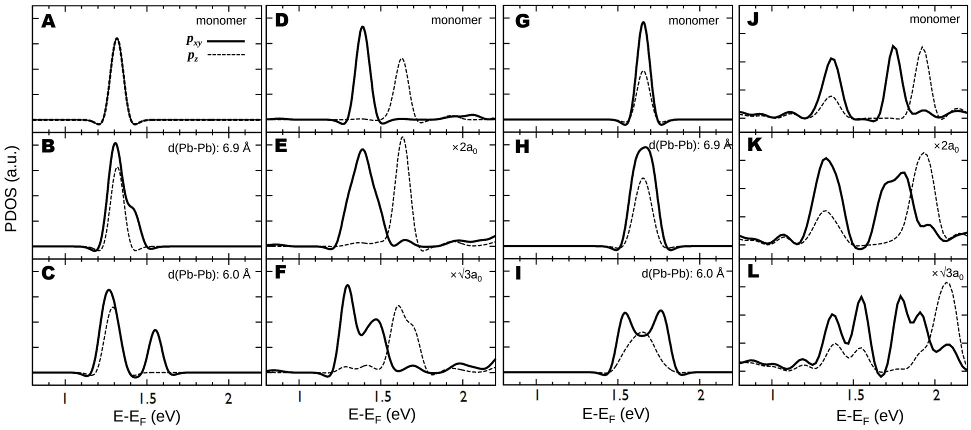

Supplementary Fig. 8: **PDOS of Pb monomer and dimers. a-c** Freestanding Pb without spin-orbit coupling (The Fermi level shifted by -1.30 eV), and **d-e** Pb on $IrTe_2$-(5×5) without spin-orbit coupling (The Fermi level shifted by -0.45 eV), and **a-c** Freestanding Pb with spin-orbit coupling (The Fermi level shifted by -0.45 eV), **g-i** Pb on $IrTe_2$-(5×5) with spin-orbit coupling (The Fermi level shifted by -0.70 eV).

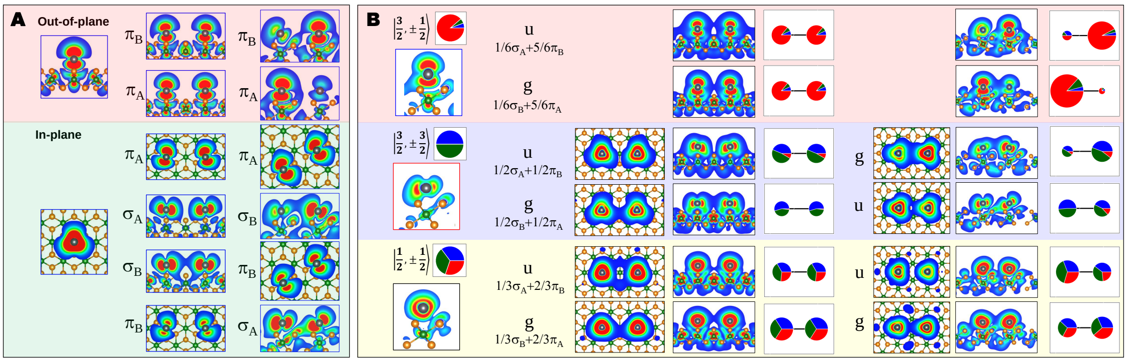

$$\left|\frac{1}{2},+\frac{1}{2}\right\rangle \propto \begin{bmatrix} -\sqrt{1/3}p_z \\ -\sqrt{1/3}\,(p_x+ip_y) \end{bmatrix} \qquad g\colon \left|\frac{1}{2},\frac{1}{2}\right\rangle - \left|\frac{1}{2},\frac{1}{2}\right\rangle \propto \begin{bmatrix} -\sqrt{1/3}p_z \\ -\sqrt{1/3}\,(p_x+ip_y) \end{bmatrix} - \begin{bmatrix} -\sqrt{1/3}p_z \\ -\sqrt{1/3}\,(p_x+ip_y) \end{bmatrix} = \begin{bmatrix} -\sqrt{1/3}\pi_z^* \\ -\sqrt{1/3}\,(\sigma_x+i\pi_y^*) \end{bmatrix}$$

$$\left|\frac{1}{2},-\frac{1}{2}\right\rangle \propto \begin{bmatrix} \sqrt{1/3}\,(p_x-ip_y) \\ -\sqrt{1/3}p_z \end{bmatrix} \qquad u\colon \left|\frac{1}{2},\frac{1}{2}\right\rangle + \left|\frac{1}{2},\frac{1}{2}\right\rangle \propto \begin{bmatrix} -\sqrt{1/3}p_z \\ -\sqrt{1/3}\,(p_x+ip_y) \end{bmatrix} + \begin{bmatrix} -\sqrt{1/3}p_z \\ -\sqrt{1/3}\,(p_x+ip_y) \end{bmatrix} = \begin{bmatrix} -\sqrt{1/3}\pi_z \\ -\sqrt{1/3}\,(\sigma_x^*+i\pi_y) \end{bmatrix}$$

$$\left|\frac{3}{2},+\frac{3}{2}\right\rangle \propto \begin{bmatrix} \sqrt{1/2}(p_x+ip_y) \\ 0 \end{bmatrix} \qquad g\colon \left|\frac{3}{2},\frac{3}{2}\right\rangle - \left|\frac{3}{2},\frac{3}{2}\right\rangle \propto \begin{bmatrix} \sqrt{1/2}(p_x+ip_y) \\ 0 \end{bmatrix} - \begin{bmatrix} \sqrt{1/2}(p_x+ip_y) \\ 0 \end{bmatrix} = \begin{bmatrix} -\sqrt{1/2}(\sigma_x+i\pi_y^*) \\ 0 \end{bmatrix}$$

$$\left|\frac{3}{2},-\frac{3}{2}\right\rangle \propto \begin{bmatrix} 0 \\ \sqrt{1/2}(p_x-ip_y) \end{bmatrix} \qquad u\colon \left|\frac{3}{2},\frac{3}{2}\right\rangle + \left|\frac{3}{2},\frac{3}{2}\right\rangle \propto \begin{bmatrix} \sqrt{1/2}(p_x+ip_y) \\ 0 \end{bmatrix} + \begin{bmatrix} \sqrt{1/2}(p_x+ip_y) \\ 0 \end{bmatrix} = \begin{bmatrix} -\sqrt{1/2}(\sigma_x^*+i\pi_y) \\ 0 \end{bmatrix}$$

$$\left|\frac{3}{2},+\frac{1}{2}\right\rangle \propto \begin{bmatrix} \sqrt{3/2}p_z \\ -\sqrt{1/6}\,(p_x+ip_y) \end{bmatrix} \qquad g\colon \left|\frac{3}{2},\frac{1}{2}\right\rangle - \left|\frac{3}{2},\frac{1}{2}\right\rangle \propto \begin{bmatrix} \sqrt{3/2}p_z \\ -\sqrt{1/6}\,(p_x+ip_y) \end{bmatrix} - \begin{bmatrix} \sqrt{3/2}p_z \\ -\sqrt{1/6}\,(p_x+ip_y) \end{bmatrix} = \begin{bmatrix} -\sqrt{3/2}\pi_z^* \\ -\sqrt{1/6}\,(\sigma_x+i\pi_y^*) \end{bmatrix}$$

$$\left|\frac{3}{2},-\frac{1}{2}\right\rangle \propto \begin{bmatrix} -\sqrt{1/6}\,(p_x+ip_y) \\ -\sqrt{3/2}p_z \end{bmatrix} \qquad u\colon \left|\frac{3}{2},\frac{1}{2}\right\rangle + \left|\frac{3}{2},\frac{1}{2}\right\rangle \propto \begin{bmatrix} \sqrt{3/2}p_z \\ -\sqrt{1/6}\,(p_x+ip_y) \end{bmatrix} + \begin{bmatrix} \sqrt{3/2}p_z \\ -\sqrt{1/6}\,(p_x+ip_y) \end{bmatrix} = \begin{bmatrix} -\sqrt{3/2}\pi_z \\ -\sqrt{1/6}\,(\sigma_x^*+i\pi_y) \end{bmatrix}$$

Supplementary Fig. 9: **Orbital characters of the Pb dimers on $IrTe_2$-(5×5) with/without spin-orbit coupling. a** without spin-orbit coupling, and **b** with spin-orbit coupling. The circles denote the angular momentum distribution (p_x: green, p_y: blue, and p_z: red) and its size is proportional to the localized state at Pb atom.

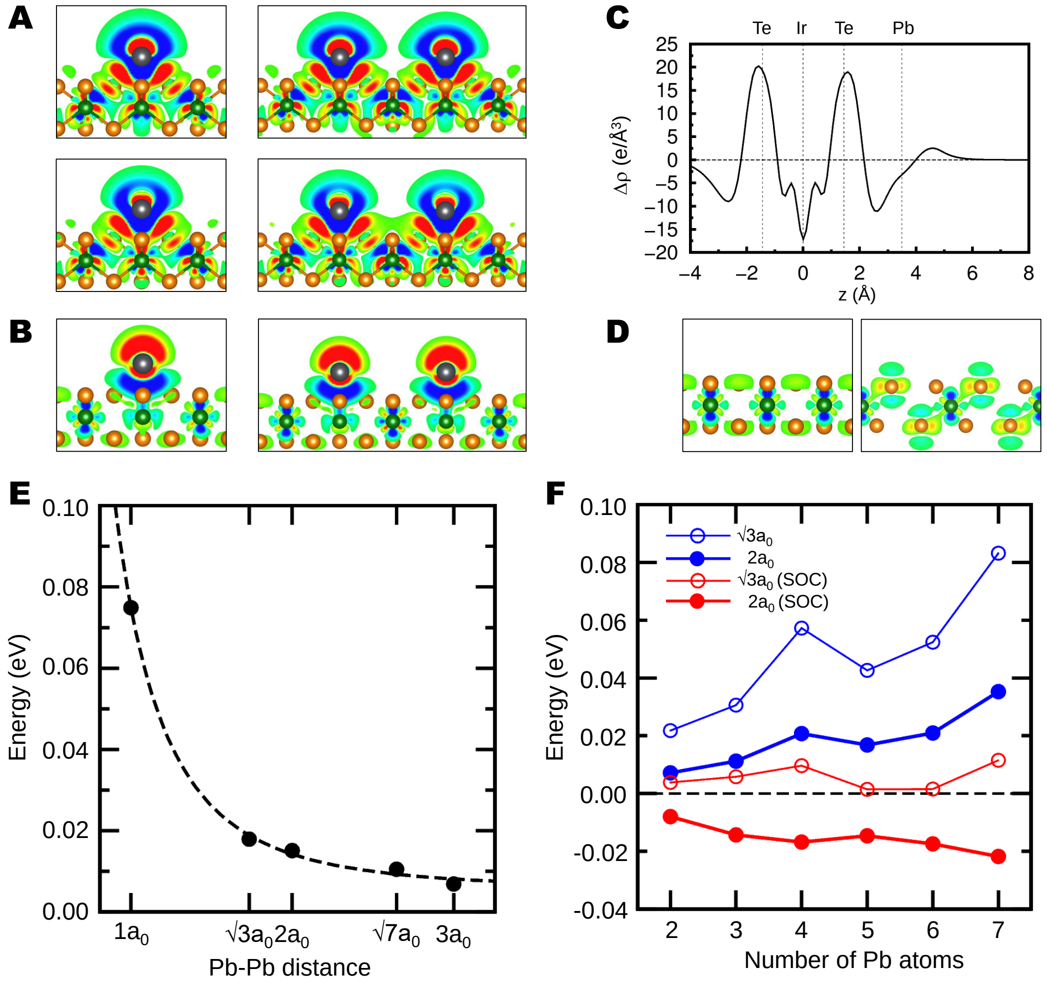

Supplementary Fig. 10: **Dipole interaction and spin-orbit coupling effect.** **a** Total charge difference ($\Delta\rho$), $\Delta\rho = \rho_{Pb/IrTe2}$ - ρ_{IrTe2} - $\rho_{Pb-atom}$. Upper and down panel are without and with spin-orbit coupling, respectively. **b** Total charge difference of Pb on $IrTe_2$ between without and with spin-orbit coupling. $\Delta\rho = \rho_{Pb/IrTe2(SOC)}$ - $\rho_{Pb/IrTe2}$. **c** In-plane summation of the charge difference. **e** Adsorption energy difference of Pb on $IrTe_2$ between without and with spin-orbit coupling. Dashed line denotes the fitting line with proportional to the 1 of r^3. **f** Relative adsorption energy of the Pb molecules including the building block with the $\sqrt{3}a_0$ spacing.

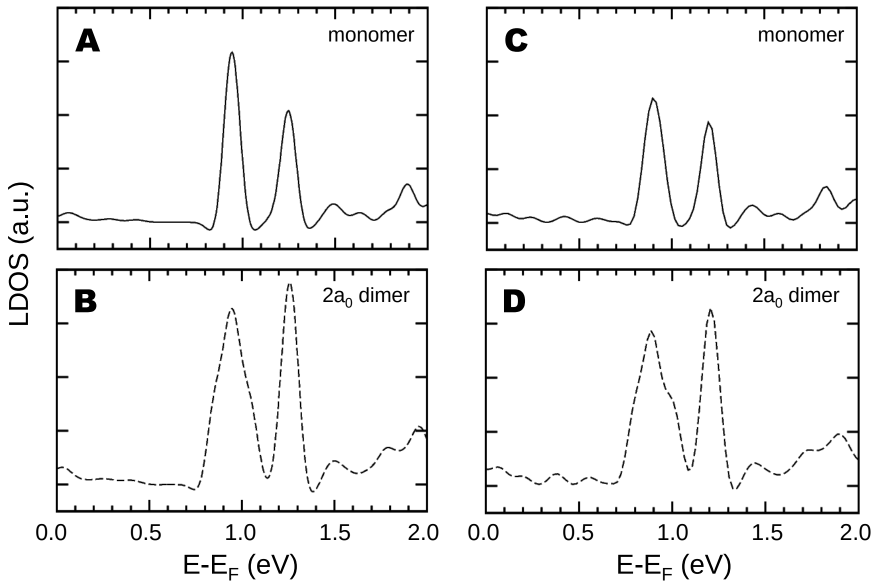

Supplementary Fig. 11: **Sn monomer and 2a$_0$ dimer on IrTe$_2$.** **a** and **b** are without spin-orbit coupling and **c** and **d** are with spin-orbit coupling. The Fermi level sets to zero.

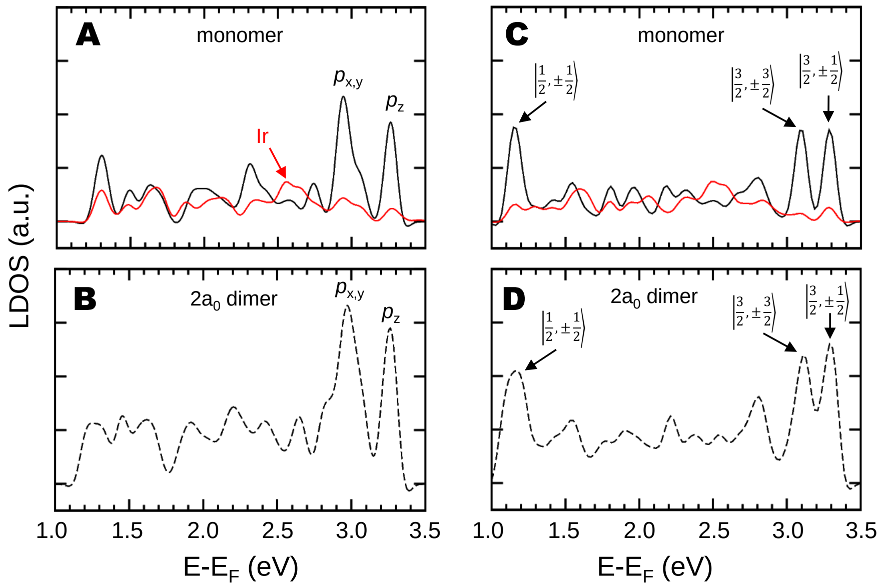

Supplementary Fig. 12: **Tl monomer and 2a$_0$ dimer on IrTe$_2$.** **a** and **b** are without spin-orbit coupling and **c** and **d** are with spin-orbit coupling. The red lines in **a** and **c** denote the localized states at Ir atom under the adsorbed Pb atom. The Fermi level sets to zero.

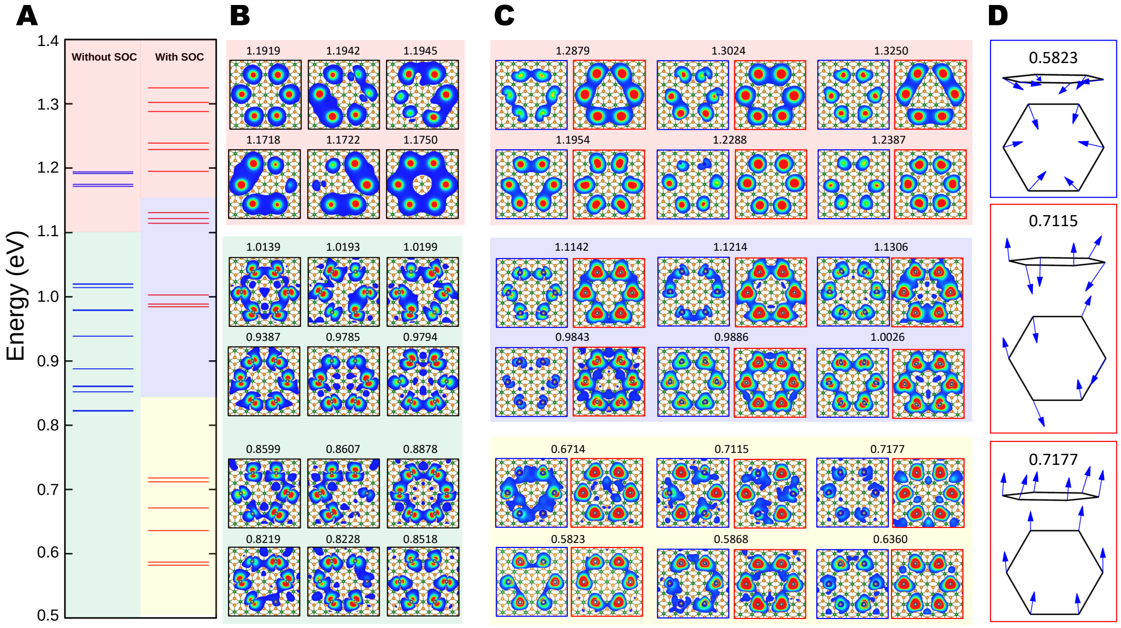

Supplementary Fig. 13: **Energy level and charge characters of the benzene-like Pb molecule on $IrTe_2$-(7×7) with/without spin-orbit coupling.** The Fermi level sets to zero. **a** The Γ point energy level for the 6*p* states of the Pb atom. **b** Charge characters without spin-orbit coupling. **c** Charge characters with spin-orbit coupling. For the case of spin-orbit coupling, there are two energetically degenerated states (red and blue) but distinguishable in their spin configuration. The number denote the corresponding energy level. **d** Selected spin configurations of rotational, anti-parallel and parallel in the $p_{1/2}$ states.

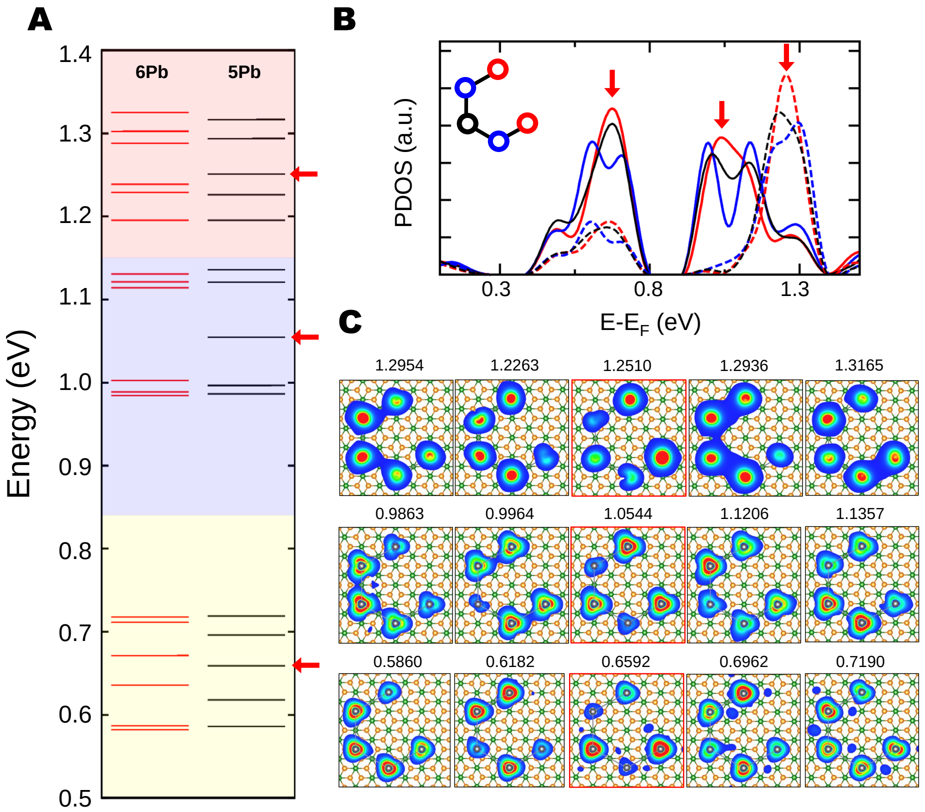

Supplementary Fig. 14: **Energy level and charge characters of the Pb pentamer on IrTe$_2$-(7×7).** **a** The Γ point energy level for the 6*p* states of the Pb atom. **b** Projected density of states. **c** Charge characters. The arrows indicate the edge states of three relativistic orbitals. The Fermi level sets to zero.

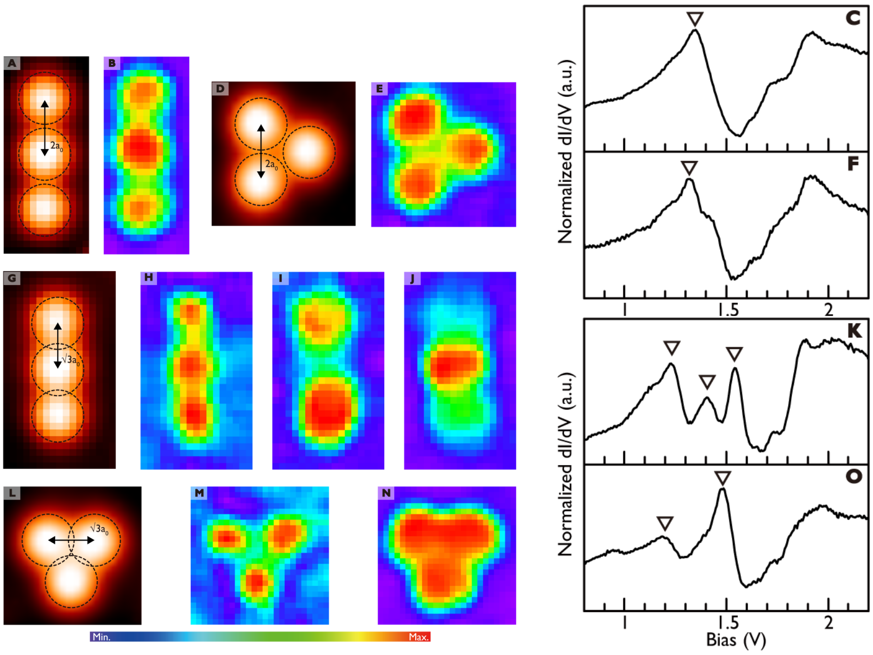

Supplementary Fig. 15: **Topography, dI/dV maps, and STS spectra of linear and triangular types of Pb trimers.** **a-c** Linear type with $2a_0$ spacing, **d-f** triangular type with $2a_0$ spacing, **g-k** linear type with $\sqrt{3}a_0$ spacing, and **l-o** triangular type with $\sqrt{3}a_0$ spacing.

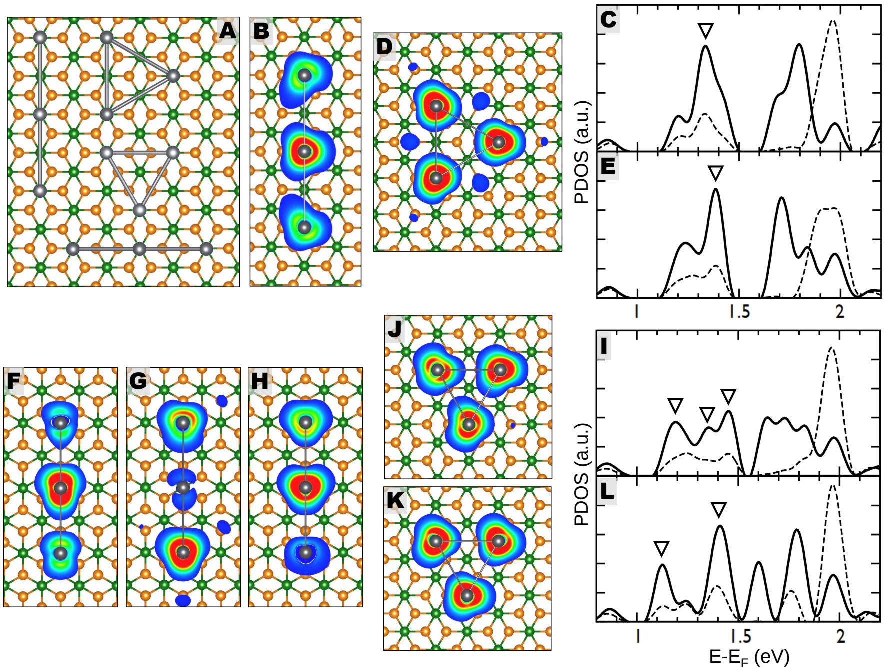

Supplementary Fig. 16: **Charge characters and PDOS of the Pb trimers on $IrTe_2$-(7×7) with spin-orbit coupling interaction.** **a** Atomic structures. **b-c** Linear type with $2a_0$ spacing, **d-e** triangular type with $2a_0$ spacing, **f-i** linear type with $\sqrt{3}a_0$ spacing, and **j-l** triangular type with $\sqrt{3}a_0$ spacing.

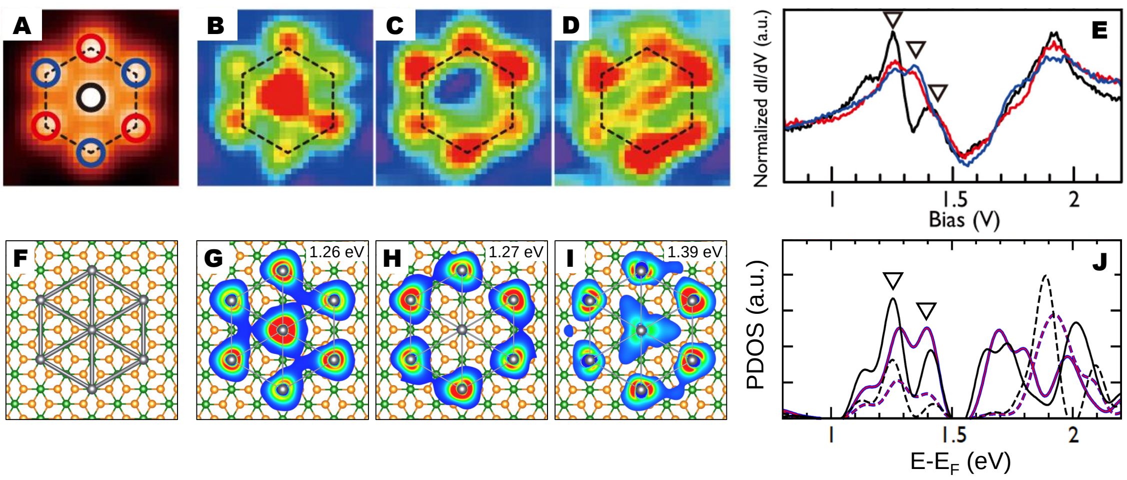

Supplementary Fig. 17: **The Pb heptamer on $IrTe_2$. a-e** Topography, dI/dV maps, and STS spectra. **f-j** Atomic structure, charge characters, and projected DOS.

Supplementary References

[1] Kim, K. *et al*. Origin of first-order-type electonic and structural transition in $IrTe_2$, *Phys. Rev. Lett.* **114**, 136401 (2015).
[2] Kim, H. S. *et al*. Nanoscale superconducting honeycomb charge order in $IrTe_2$, *Nano Lett.* **16**, 4260 (2016).
